# End point prick test: could this new test be used to predict the outcome of oral food challenge in children with cow's milk allergy?

**DOI:** 10.1186/1824-7288-37-52

**Published:** 2011-11-04

**Authors:** Federica Bellini, Giampaolo Ricci, Arianna Dondi, Valentina Piccinno, Federico Angelini, Andrea Pession

**Affiliations:** 1 Pediatric Unit, Department of Gynecologic, Obstetric and Pediatric Sciences, University of Bologna, Bologna, Italy

**Keywords:** Cow's milk proteins allergy, end point prick test, food oral challenge, tolerance

## Abstract

**Background:**

Cow's milk allergy (CMA) is the most frequent food allergy in childhood; the trend of CMA is often characterized by a progressive improvement to achieve tolerance in the first 4 to 5 years of life.

It has been observed that specific IgE (sIgE) towards cow's milk proteins decrease when the age increases.

Although food allergy can be easily diagnosed, it is difficult to predict the outcome of the oral food challenge (OFC), that remains the gold standard in the diagnosis of food allergy, by allergometric tests.

**Methods:**

We considered 44 children with CMA diagnosed through OFC who returned to our Allergy and Immunology Pediatric Department between January to December 2010 to evaluate the persistence of allergy or the achievement of tolerance.

On the basis of the history, we performed both allergometric skin tests and OFC in children that were still following a milk-free diet, whereas only allergometric skin tests those that had already undergone spontaneous introduction of milk protein at home without presenting symptoms.

**Objective:**

The aim of this study was to investigate the relationship between the persistence of CMA or the acquisition of tolerance and the results of the end point prick test (EPT).

**Results and Discussion:**

The OFC with cow's milk was performed on 30 children, 4 children were excluded because of a history of severe reactions to cow's milk, and 10 because they had spontaneously already taken milk food derivates at home without problems. 16/30 (53%) children showed clinical reactions and the challenge was stopped, 14/30 (47%) did not have any reaction.

Comparing the mean wheal diameter of every EPT's dilution between the group of allergic children and the tolerant ones, we obtained a significant difference (p < 0.05) for the first 4 dilutions.

We have also calculated sensitivity (SE), specificity (SP), the positive predictive value (PPV) and the negative predictive value (NPV) for each EPT dilution.

**Conclusions:**

EPT is a safe and cheap test, easy to be executed and that could provide good prediction of the outcome of OFC; so it might be used to avoid OFC-induced anaphylaxis in children affected by CMA. It can also help avoiding dietetic restrictions in tolerant children who show sensitization towards cow's milk proteins.

## Background

Despite the risk of anaphylaxis in allergic children, the OFC is considered the gold standard for diagnosing CMA. It confirms the suspicion of CMA, it helps monitoring the resolution of CMA and its follow up and it evaluates the necessity of dietary restriction [1-6].

Many authors have tried to correlate the skin prick test (SPT) results or the specific IgE (sIgE) levels to the outcome of OFC [7-14], in order to find a cheap and safe test with a good prediction, to reduce the economic costs and avoid the risks of anaphylaxis related to the OFC. Sporik et al. [10] and Calvani et al. [11] for instance tried to correlate the SPT wheal diameters obtained with cow's milk and with some proteic fractions of milk to the outcome of OFC, but they found different cut-off values, sensitivity was not high enough to prevent allergic reactions during the OFC in allergic children, and moreover wheal diameters measurement in SPT were influenced by the operator.

Sporik et al. [10] identified for each food a skin wheal diameter cut-off above which negative reactions did not occur: in particular, for cow's milk the wheal cut-off was 8 mm. In contrast, positive reactions could occur with a negative test.

Calvani et al. [11] compared the diagnostic capacity of SPTs for the three main cow's milk proteins (a-lactalbumin, casein and b-lactoglobulin) with fresh milk and tried to determine a cut-off to discriminate between allergic and tolerant children in a controlled OFC. They showed that having positive SPTs for all three cow's milk proteins had PPV of 92.3%, being clinically more useful than any cut-off. The positivity of SPT to all three cow's milk proteins seems to be a simpler and more useful way of avoiding OFCs.

Correlations between milk proteins sIgE levels and the outcome of OFC can be found in many papers [12-15]. Anyway the parameters to predict the challenge outcome vary by the children age, by the proteic fractions considered and by the measuring methodics.

Another cutaneous test, the EPT has been considered for a long time as an accurate method of classifying sensitivity to many allergens during the 70s, but only recently it has been included in food allergy diagnosis [15,16].

EPT with cow's milk consists of a SPT with raw milk and seven progressive dilutions (1D: 1/10, 2D: 1/100, 3D: 1/1.000, 4D: 1/10.000, 5D: 1/100.000, 6D: 1/1.000.000, 7D: 1/10.000.000).

A recent work by Mori et al. [16] used this test to determine the starting dose of oral desensitization.

## Methods

### Subjects in the study

We considered 44 children with CMA diagnosed through OFC who returned to our Allergy and Immunology Pediatric Department between January to December 2010 to evaluate the persistence of allergy or the achievement of tolerance. The mean age of the 44 children with CMA at diagnosis was 6 months; 18 (42%) children were males, 26 (58%) females.

At onset, 31/44 (70.5%) patients presented cutaneous symptoms, namely generalized urticaria-angioedema in 18 (58%) and contact urticaria associated with AD in 13 (42%); 9/44 (20.4%) developed gastrointestinal symptoms (3 vomiting, 2 abdominal pain and/or diarrhoea, 4 vomiting and diarrhea); 4/44 (9.1%) had anaphylaxis.

### Plan

On the basis of the history, we performed both allergometric skin tests and OFC in children that were still following a milk-free diet, whereas only allergometric skin tests in those that had already undergone spontaneous introduction of milk protein at home without presenting symptoms.

### Skin Prick Test

In all 44 children SPT was performed with fresh cow's milk and the commercial milk extract (Lofarma, Italy). The positive control was carried out with a histamine standard (1 mg/ml) and a negative control with a glycerosaline solution. A wheal reaction ≥ 3 mm was required for positivity.

### End Point Test

EPT consists of seven progressive dilutions of fresh cow's milk (30 mg/ml) with saline solution (1D: 1/10 = 3 mg/ml, 2D: 1/100 = 0.3 mg/ml, 3D: 1/1.000 = 0.03 mg/ml, 4D: 1/10.000 = 0.003 mg/ml, 5D: 1/100.000 = 0.0003 mg/ml, 6D: 1/1.000.000 = 0.00003 mg/ml, 7D: 1/10.000.000 = 0.000003 mg/ml) in 10 ml plastic tubes. For the dilution 1:10 we added 9 ml of saline solution to 1 ml of fresh milk. To obtain the dilution 1:100 we added 9 ml of saline solution to 1 ml drawn out from the 1:10 dilution and so on. EPT were performed on the same day of SPT by the same investigator on the volar surface of the forearm. We considered a wheal reaction ≥ 2 mm as positive.

### Specific IgE

The determination of cow's milk sIgE was performed by ImmunoCAP™ (Phadia, Sweden). Values greater than 0.10 kUa/L were considered as positive.

### Oral Food Challenge

We started the challenge with 1 drop of cow's milk, then we progressively increased every 20 minutes the amount of milk administered according to this scheme: 1 ml, 5 ml, 10 ml, 20 ml, 40 ml, 50 ml, 100 ml. The challenge was stopped in case of clinical reactions. During OFC children were completely free from any treatment with antihistamines. Children that had not experienced clinical reactions during the challenge were defined tolerant, whereas those who presented clinical reactions were defined allergic. On the basis of the outcome of the OFC, allergic patients maintained an exclusion diet, contrarily to tolerant patients who were allowed to include milk in their diet.

### Statistical analysis

Statistical analysis was performed by means of SPSS 15 for Windows, SPSS Inc, Chicago, Ill. Student's *t*-test was used for the comparison of mean values. Probability values of less than 0.05 were considered as statistically significant. Two by two tables were used to calculate sensitivity (SE), specificity (SP), positive predictive value (PPV) and negative predictive value (NPV). SE was defined as the proportion of true positives detected, specificity as the proportion of true negatives detected. PPV describes the proportion of the true positives among the apparent positives, while NPV shows the proportion of the true negatives among the apparent negatives. Candlestick charts were used to compare the same parameters in different groups of patients. Predictive decision points for a positive OFC were calculated through receiver operating characteristic (ROC) analysis. The Geometric Mean of sIgE levels was calculated considering the average of the logarithmic values converted back to a base 10 number.

### Ethics

This retrospective study was only observational and did not interfere with the clinical management of the patients, so it was no submitted to the ethical committee for approval. However an informed consent was signed by parents of each patient before starting the OFC. The consent of challenge was approved in November 2005 by the legal and quality commission of University Hospital - S.Orsola-Malpighi of Bologna.

## Results

All 44 children had positive SPT both for fresh milk (mean diameter of the wheal 9.7 mm) and for the commercial extract (mean diameter 5.2 mm).

The OFC with cow's milk was performed on 30 children, 4 children were excluded because of a history of severe reactions to cow's milk, and 10 because they had spontaneously already taken milk food derivates at home without problems. 16/30 (53%) children (mean age 18 months) showed clinical reactions and the challenge was stopped, 14/30 (47%) children (mean age 24.4 months) did not have any reactions.

Comparing the mean wheal diameter of every EPT dilution between the group of allergic children and the tolerant ones with Student's t test, we obtained a s.s. difference (p < 0.05) for the first 4 dilutions (Table 1).

**Table 1:** Percentage of patients positive to EPT: comparison between allergic and tolerant children.

	Allergic (%)	Mean wheal diameter (mm)	Tolerant (%)	Mean wheal diameter (mm)
Fresh milk	**20 (100%)**	9.7*	**24 (100%)**	5.2*

1D	**20 (100%)**	7.6*	**24 (100%)**	4.2*

2D	**19/20(95%)**	6.3*	**19/24 (78%)**	3.2*

3D	**18/20 (90%)**	4.7*	**11/24 (42%)**	2.5*

4D	**18/20 (50%)**	4.5*	**3/24 (12%)**	2.2*

5D	**6/20 (30%)**	3	**1/24 (4,2%)**	2.5

6D	**2/20 (11%)**	2.5	**0/24 (0%)**	0

7D	**2/20 (11%)**	2.5	**0/24 (0%)**	0

*P value < 0.05

Furthermore we calculated the SE, the SP, PPV and the NPV of the 7 dilutions (Table 2).

**Table 2:** EPT: Sensitivity (SE), specificity (SP), positive predictive value (PPV) and negative predictive value (EPV) of each dilution.

	SE	SP	PPV	NPV
1D	100%	-	45,5%	-

2D	95%	21%	50%	83%

3D	90%	54%	62%	87%

4D	50%	87,5%	86%	91%

5D	30%	96%	86%	62%

6D	11%	100%	100%	57%

7D	11%	100%	100%	57%

In particular we found that the 4D EPT (SE 50%, SP 87.5%, PPV 86%, NPV 91%) has the best ratio between PPV and NPV.

**Figure 1:**
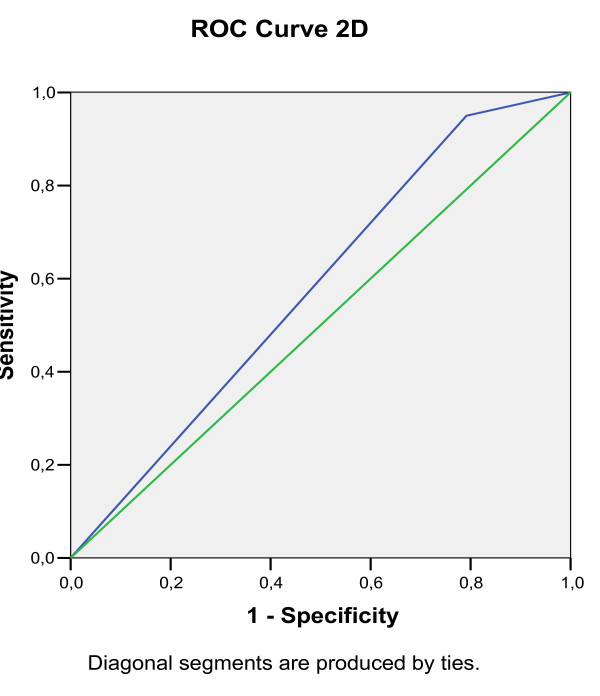
**Receiver operating characteristic (ROC) analysis of a positive OFC, calculated for 2D EPT dilution**.

**Figure 2:**
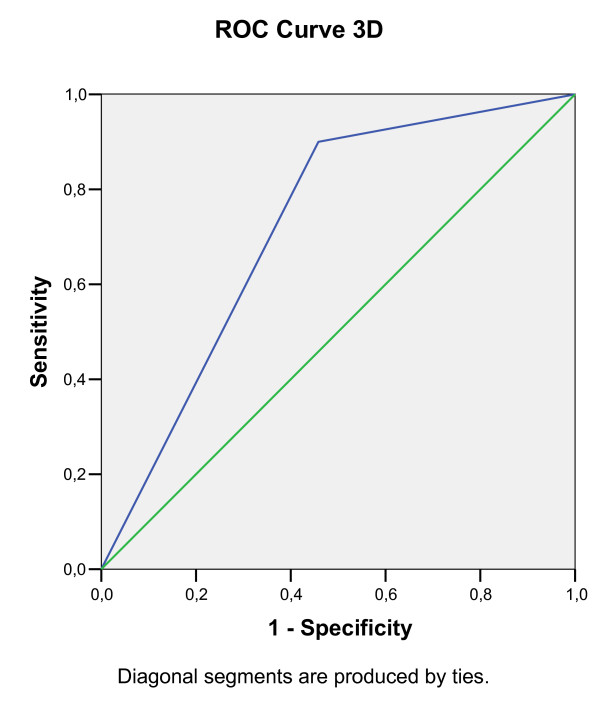
**Receiver operating characteristic (ROC) analysis of a positive OFC, calculated for 3D EPT dilution**.

**Figure 3:**
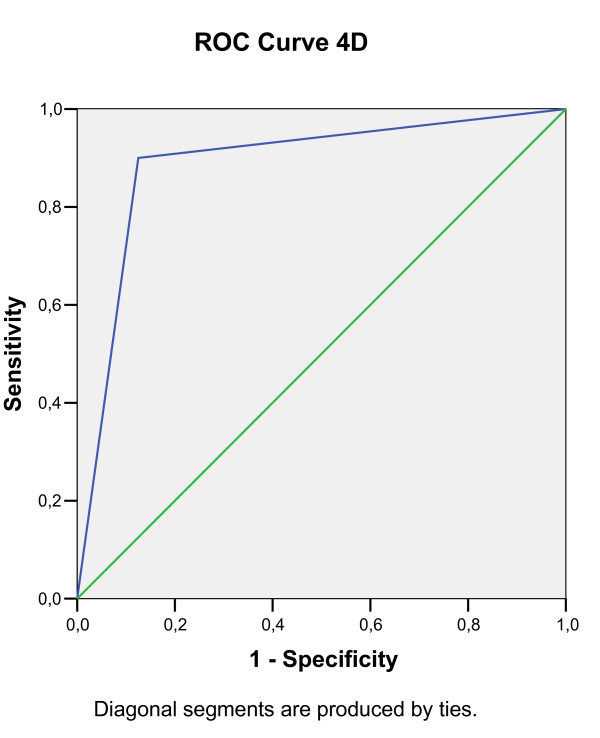
**Receiver operating characteristic (ROC) analysis of a positive OFC, calculated for 4D EPT dilution**. Area under the curve: 0.88. The test result variable(s): 4D test has at least one tie between the positive actual state group and the negative actual state group.

**Figure 4:**
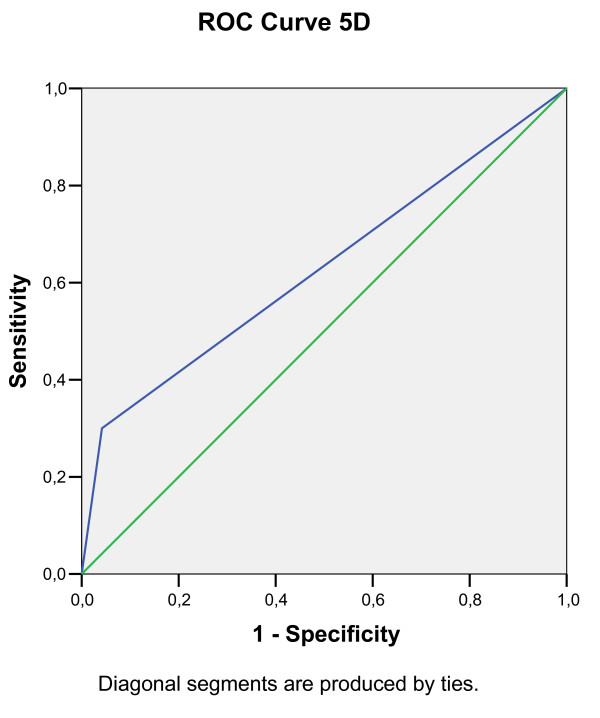
**Receiver operating characteristic (ROC) analysis of a positive OFC, calculated for 5D EPT dilution**.

**Figure 5:**
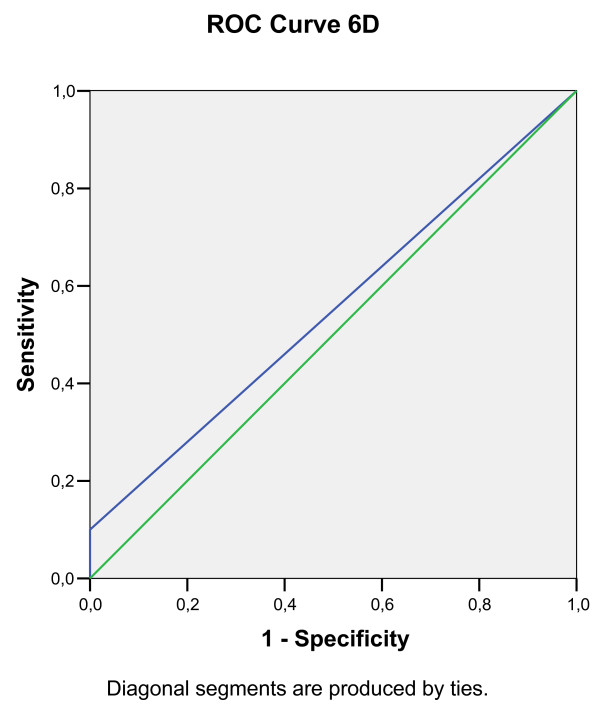
**Receiver operating characteristic (ROC) analysis of a positive OFC, calculated for 6D EPT dilution**.

**Figure 6:**
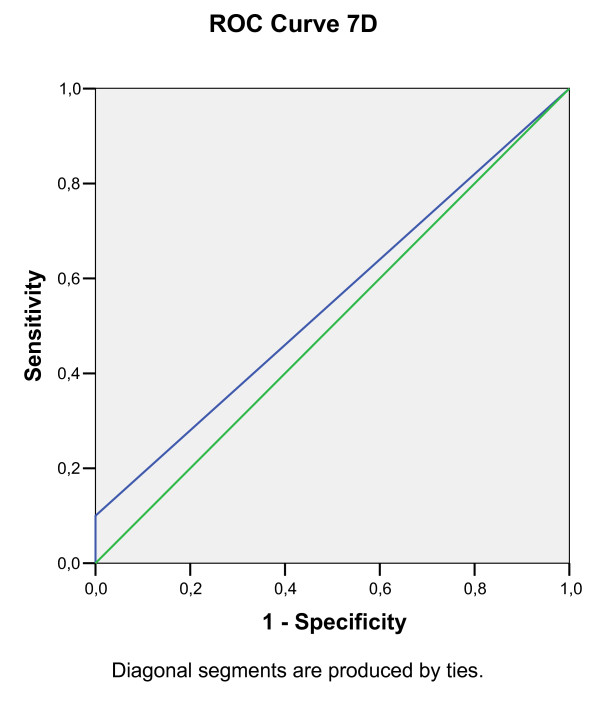
**Receiver operating characteristic (ROC) analysis of a positive OFC, calculated for 7D EPT dilution**.

Predictive decision points for a positive OFC were calculated through receiver operating characteristic (ROC) analysis (Figure 1, 2, 3, 4, 5, 6).

In particular ROC analysis calculated for 4D EPT (Figure 3) shows that area under the curve is 0.88 and that 4D EPT has at least one tie between the positive actual state group and the negative actual state group. Furthermore we calculated the Geometric means of sIgE levels against cow's milk in both tolerant and allergic patients (sIgE Geometric mean 6.15 kU/l in the first group and 38.2 kU/l in the second one, *P < 0.05*).

## Conclusions

Symptoms of food allergy vary greatly and diagnosis is not always easy to make; the OFC is currently the gold standard to diagnose food allergy. It can be actually a risky test, it is expensive, and moreover there are no clear guidelines to choose which children should be tested and which not. Objective parameters correlated to challenge outcome are missing.

Calvani et al. [11] showed that a SPT positive for all three milk proteins had a PPV of 92.3% and would seem more clinically useful than any cut-off. The positivity of SPT to all three cow's milk proteins seems to be a simpler and more useful way of avoiding OFCs. Mori et al. [16] used EPT to calculate the first dose for oral desensitization: all children started the OFC with the dilution immediately below the positive one and it resulted that: 6.7% patients had the threshold concentration at 1:100.000, 33.3% at 1:10.000, 43.3% at 1:1000, 13.3% at 1:100 and 3.3% at 1:10. They concluded that EPT allows to be more confident with each single child, reducing the risk of reaction at the beginning.

In this study we found out that EPT is a safe test and that being positive to the 4D could be the first step after a positive SPT to cow's milk to select children who should not try OFC.

The 4D allows to discriminate the highest number of allergic patients from the tolerant ones, with a better ratio between PPV and NPV.

A negative EPT to 4D does not show a high predictive meaning: 50% of negative children show reactions during OFC.

Considering the prevalence of the disease in our cohort's patients, we obtained a Likelihood Ratio (LHR) = 4, but these date maybe greatly different if we consider a general population with a lower prevalence around 20%, in fact in this condition, the post-test probability, using the Fagan nomogram, falls down to 50%.

Moreover every dilution of EPT has its statistical significance, and could help showing border line children situations. EPT is also a safe and cheap test, easily performed without risk of adverse reactions.

Should the usefulness of EPT be confirmed, this test might replace OFC avoiding any risk to children affected by food allergy and could be a valid approach to improve the use of the skin test and predict the outcome of the OFC.

## Competing interests

The authors declare that they have no competing interests.

## Authors' contributions

FB: data collection, data analysis, literature search, abstract presentation, manuscript preparation. GR: planning, study design and manuscript preparation. AD: data collection, data analysis, literature search. VP and FA: data analysis, literature search. AP: supervision and manuscript preparation. All authors read and approved the final manuscript.
